# The defect scheelite-type lanthanum(III) *ortho*-oxidomolybdate(VI) La_0.667_[MoO_4_]

**DOI:** 10.1107/S1600536813000731

**Published:** 2013-01-16

**Authors:** Tanja Schustereit, Thomas Schleid, Ingo Hartenbach

**Affiliations:** a Institut für Anorganische Chemie, Universität Stuttgart, Pfaffenwaldring 55, 70569 Stuttgart, Germany

## Abstract

In scheelite-type La_0.667_[MoO_4_], one crystallographically unique position with site symmetry -4.. and an occupancy of 2/3 is found for the La^3+^ cation. The cation is surrounded by eight O atoms in the shape of a trigonal dodeca­hedron. The structure also contains one [MoO_4_]^2−^ anion (site symmetry -4..), which is surrounded by eight vertex-attached La^3+^ cations. The polyhedra around the La^3+^ cations are inter­connected *via* common edges, building up a three-dimensional network, in the tetra­hedral voids of which the Mo^6+^ cations reside.

## Related literature

For isotypic *Ln*_0.667_[MoO_4_] structures with *Ln* = Ce, Pr, Nd and Sm, see: Schustereit *et al.* (2011 ▶). For synthetic details, see: Liu *et al.* (2012 ▶).

## Experimental

### 

#### Crystal data

La_0.667_[MoO_4_]
*M* *_r_* = 252.59
Tetragonal, 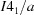

*a* = 5.3599 (3) Å
*c* = 11.9425 (7) Å
*V* = 343.09 (3) Å^3^
*Z* = 4
Mo *K*α radiation
μ = 11.74 mm^−1^
*T* = 293 K
0.11 × 0.08 × 0.06 mm

#### Data collection

Nonius KappaCCD diffractometer
Absorption correction: numerical (*X-SHAPE*; Stoe & Cie, 1995 ▶) *T*_min_ = 0.291, *T*_max_ = 0.480
2577 measured reflections
260 independent reflections
174 reflections with *I* > 2σ(*I*)
*R*_int_ = 0.046

#### Refinement

*R*[*F* ^2^ > 2σ(*F* ^2^)] = 0.016
*wR*(*F* ^2^) = 0.032
*S* = 0.98
260 reflections
16 parameters
Δρ_max_ = 0.41 e Å^−3^
Δρ_min_ = −0.37 e Å^−3^

### 

Data collection: *COLLECT* (Nonius, 1998 ▶); cell refinement: *SCALEPACK* (Otwinowski & Minor, 1997 ▶); data reduction: *SCALEPACK* and *DENZO* (Otwinowski & Minor, 1997 ▶); program(s) used to solve structure: *SHELXS97* (Sheldrick, 2008 ▶); program(s) used to refine structure: *SHELXL97* (Sheldrick, 2008 ▶); molecular graphics: *DIAMOND* (Brandenburg, 2006 ▶); software used to prepare material for publication: *SHELXL97*.

## Supplementary Material

Crystal structure: contains datablock(s) I, global. DOI: 10.1107/S1600536813000731/wm2715sup1.cif

Structure factors: contains datablock(s) I. DOI: 10.1107/S1600536813000731/wm2715Isup2.hkl

Additional supplementary materials:  crystallographic information; 3D view; checkCIF report

## Figures and Tables

**Table 1:** Selected bond lengths (Å)

La—O^i^	2.5728 (14)
La—O^ii^	2.5766 (14)
Mo—O	1.7606 (14)

Symmetry codes: (i) 
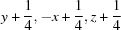
; (ii) 
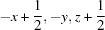
.

**Fig. 1.**
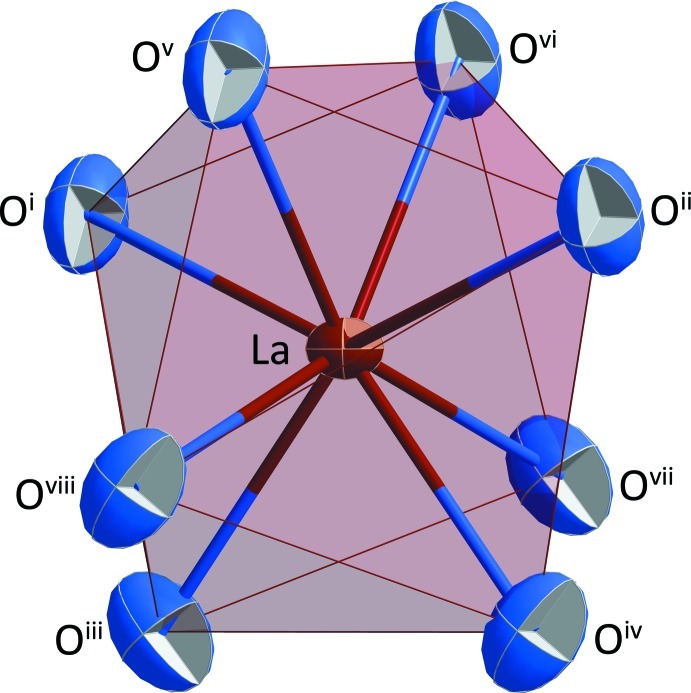
Trigonal dodecahedral oxygen environment of the La3+ cation in defect scheelite-type La0.667[MoO4]; ellipsoids are drawn at the 80 % probability level. [Symmetry codes: (i) x, y, z; (ii) –x+1/2, –y, z+1/2; (iii) –y+3/4, x+1/4, z+1/4; (iv) y+3/4, –x+3/4, z+3/4; (v) –x, –y, –z; (vi) x+1/2, y, –z+1/2; (vii) y+1/4, –x+3/4, –z+3/4; (viii) –y+1/4, x+1/4, –z+1/4].

**Fig. 2.**
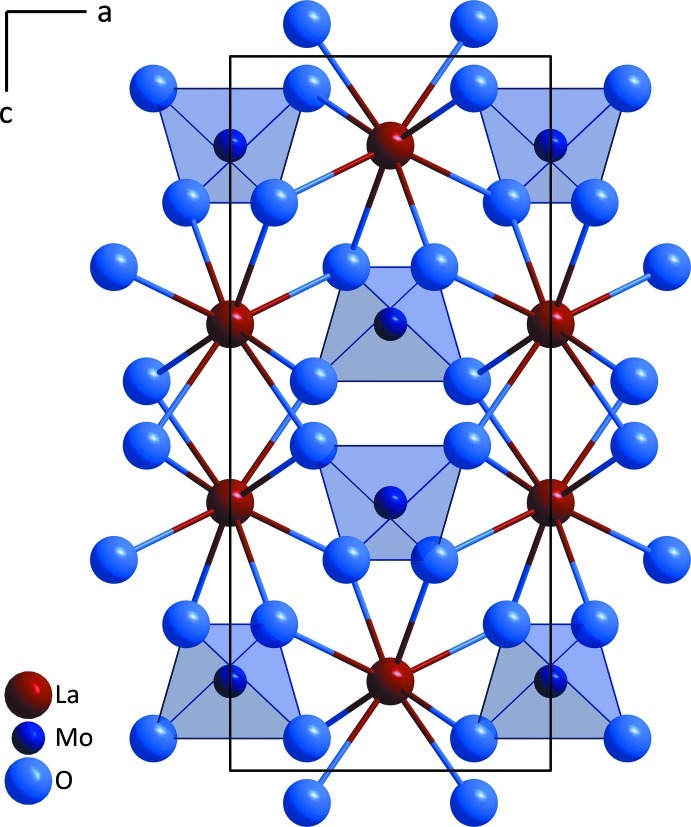
View of the crystal structure of defect scheelite-type La0.667[MoO4] along [010]; the bisphenoidally distorted tetrahedral ortho-oxidomolybdate(VI) units [MoO4]2– are given in the polyhedral representation.

## supplementary crystallographic information

### Comment

The title compound crystallizes isotypically with the already known defect scheelite-type lanthanide ortho-oxidomolybdates(VI) with general formula *Ln*_0.667_[MoO_4_] (*Ln* = Ce, Pr, Nd, and Sm; Schustereit *et al.*, 2011). The crystallographically unique La^3+^ cation at Wyckoff position 4*b* is surrounded by eight oxide anions in the shape of a trigonal dodecahedron (Fig. 1). Besides the lanthanum cation, the structure also contains an isolated and bisphenoidally distorted tetrahedral ortho-oxidomolybdate(VI) unit [MoO_4_]^2–^. Its central Mo^6+^ cation at Wyckoff position 4*a* exhibits the same site symmetry (4..) as the La^3+^ cation. Hence, in order to maintain electroneutrality, the latter shows a statistically under-occupation of 2/3 on its atomic position. The [MoO_4_]^2–^ tetrahedra share common vertices with eight [LaO_8_]^13–^ dodecahedra to build up the scheelite-type crystal structure (Fig. 2). The cations at the sites 4*a* (La^3+^) and 4*b* (Mo^6+^) are thereby arranged in two interpenetrating diamond-like lattices (Schustereit *et al.*, 2011).

### Experimental

Colourless, irregular-shaped single crystals of scheelite-type La_0.667_[MoO_4_] were obtained as a by-product in an unsuccessful attempt to synthesize LaF[MoO_4_] according to the Pecchini method (Liu *et al.*, 2012). Aqueous solutions with stoichiometric amounts of La(NO_3_)_3_ and (NH_4_)_6_Mo_7_O_24_ (molar ratio 7 : 1) were prepared for each compound and HF was added to the latter (molar ratio HF : (NH_4_)_6_Mo_7_O_24_ = 7 : 1). As chelating agent, citric acid (CA) was dissolved in both solutions with a molar ratio of CA : La^3+^ = 1 : 1 and CA : Mo^6+^ = 2 : 1. The pH value of the La^3+^-containing solution was adjusted to 3 – 4 with an aqueous ammonia solution as well as the pH value of the CA/HF/(NH_4_)_6_Mo_7_O_24_ mixture, in this case to a value of 7 – 8. The two solutions were combined, stirred, and heated for about 30 minutes to obtain a transparent solution, which was then dried at 473 K for 5 hours. The residual product was thermally treated in air at 1123 K for 12 hours.

### Refinement

The site occupation factor of the La^3+^ site was refined freely to a value of 0.6676 (10).

### Crystal data

**Table:**

La_0.667_[MoO_4_]	*D*_x_ = 4.890 Mg m^−^^3^
*M**_r_* = 252.59	Mo *K*α radiation, λ = 0.71073 Å
Tetragonal, *I*4_1_/*a*	Cell parameters from 1769 reflections
Hall symbol: -I 4ad	θ = 0.4–30.5°
*a* = 5.3599 (3) Å	µ = 11.74 mm^−^^1^
*c* = 11.9425 (7) Å	*T* = 293 K
*V* = 343.09 (3) Å^3^	Irregular, colourless
*Z* = 4	0.11 × 0.08 × 0.06 mm
*F*(000) = 448	

### Data collection

**Table:**

Nonius KappaCCD diffractometer	260 independent reflections
Radiation source: fine-focus sealed tube	174 reflections with *I* > 2σ(*I*)
Graphite monochromator	*R*_int_ = 0.046
ω and φ scans	θ_max_ = 30.4°, θ_min_ = 4.2°
Absorption correction: numerical (*X-SHAPE*; Stoe & Cie, 1995)	*h* = −7→7
*T*_min_ = 0.291, *T*_max_ = 0.480	*k* = −7→7
2577 measured reflections	*l* = −16→16

### Refinement

**Table:**

Refinement on *F*^2^	Primary atom site location: structure-invariant direct methods
Least-squares matrix: full	Secondary atom site location: difference Fourier map
*R*[*F*^2^ > 2σ(*F*^2^)] = 0.016	*w* = 1/[σ^2^(*F*_o_^2^) + (0.0079*P*)^2^] where *P* = (*F*_o_^2^ + 2*F*_c_^2^)/3
*wR*(*F*^2^) = 0.032	(Δ/σ)_max_ < 0.001
*S* = 0.98	Δρ_max_ = 0.41 e Å^−^^3^
260 reflections	Δρ_min_ = −0.37 e Å^−^^3^
16 parameters	Extinction correction: *SHELXL97* (Sheldrick, 2008), Fc^*^=kFc[1+0.001xFc^2^λ^3^/sin(2θ)]^-1/4^
0 restraints	Extinction coefficient: 0.0082 (5)

### Special details

**Table:**

Geometry. All esds (except the esd in the dihedral angle between two l.s. planes) are estimated using the full covariance matrix. The cell esds are taken into account individually in the estimation of esds in distances, angles and torsion angles; correlations between esds in cell parameters are only used when they are defined by crystal symmetry. An approximate (isotropic) treatment of cell esds is used for estimating esds involving l.s. planes.
Refinement. Refinement of F^2^ against ALL reflections. The weighted R-factor wR and goodness of fit S are based on F^2^, conventional R-factors R are based on F, with F set to zero for negative F^2^. The threshold expression of F^2^ > 2sigma(F^2^) is used only for calculating R-factors(gt) etc. and is not relevant to the choice of reflections for refinement. R-factors based on F^2^ are statistically about twice as large as those based on F, and R- factors based on ALL data will be even larger.

### Fractional atomic coordinates and isotropic or equivalent isotropic displacement parameters (Å^2^)

**Table:**

	*x*	*y*	*z*	*U*_iso_*/*U*_eq_	Occ. (<1)
La	0.0000	0.2500	0.6250	0.01492 (18)	0.6676 (10)
Mo	0.0000	0.2500	0.1250	0.01706 (15)	
O	0.1374 (3)	0.0106 (2)	0.20490 (13)	0.0313 (5)	

### Atomic displacement parameters (Å^2^)

**Table:**

	*U*^11^	*U*^22^	*U*^33^	*U*^12^	*U*^13^	*U*^23^
La	0.0171 (2)	0.0171 (2)	0.0107 (2)	0.000	0.000	0.000
Mo	0.01534 (18)	0.01534 (18)	0.0205 (2)	0.000	0.000	0.000
O	0.0209 (8)	0.0329 (9)	0.0403 (9)	0.0048 (6)	0.0031 (9)	0.0105 (7)

### Geometric parameters (Å, º)

**Table:**

La—O^i^	2.5728 (14)	La—La^x^	4.0120 (2)
La—O^ii^	2.5728 (14)	La—La^xi^	4.0120 (2)
La—O^iii^	2.5728 (14)	La—La^iv^	4.0120 (2)
La—O^iv^	2.5728 (14)	Mo—O^xii^	1.7605 (14)
La—O^v^	2.5766 (14)	Mo—O^xiii^	1.7605 (14)
La—O^vi^	2.5766 (14)	Mo—O^xiv^	1.7605 (14)
La—O^vii^	2.5766 (14)	Mo—O	1.7606 (14)
La—O^viii^	2.5766 (14)	O—La^iv^	2.5728 (14)
La—La^ix^	4.0120 (2)	O—La^xv^	2.5766 (14)
			
O^i^—La—O^ii^	128.57 (4)	O^iii^—La—La^x^	161.84 (3)
O^i^—La—O^iii^	75.70 (7)	O^iv^—La—La^x^	66.64 (3)
O^ii^—La—O^iii^	128.57 (4)	O^v^—La—La^x^	103.20 (4)
O^i^—La—O^iv^	128.57 (4)	O^vi^—La—La^x^	38.79 (3)
O^ii^—La—O^iv^	75.70 (7)	O^vii^—La—La^x^	129.62 (3)
O^iii^—La—O^iv^	128.57 (4)	O^viii^—La—La^x^	85.04 (3)
O^i^—La—O^v^	148.98 (6)	La^ix^—La—La^x^	123.628 (3)
O^ii^—La—O^v^	68.24 (3)	O^i^—La—La^xi^	161.84 (3)
O^iii^—La—O^v^	74.21 (2)	O^ii^—La—La^xi^	66.64 (3)
O^iv^—La—O^v^	77.64 (5)	O^iii^—La—La^xi^	103.00 (3)
O^i^—La—O^vi^	74.21 (2)	O^iv^—La—La^xi^	38.85 (3)
O^ii^—La—O^vi^	77.64 (5)	O^v^—La—La^xi^	38.79 (3)
O^iii^—La—O^vi^	148.98 (6)	O^vi^—La—La^xi^	103.20 (4)
O^iv^—La—O^vi^	68.24 (3)	O^vii^—La—La^xi^	85.04 (3)
O^v^—La—O^vi^	136.53 (7)	O^viii^—La—La^xi^	129.62 (3)
O^i^—La—O^vii^	77.64 (5)	La^ix^—La—La^xi^	123.628 (3)
O^ii^—La—O^vii^	148.98 (6)	La^x^—La—La^xi^	83.823 (4)
O^iii^—La—O^vii^	68.24 (3)	O^i^—La—La^iv^	38.85 (3)
O^iv^—La—O^vii^	74.20 (2)	O^ii^—La—La^iv^	161.84 (3)
O^v^—La—O^vii^	97.88 (2)	O^iii^—La—La^iv^	66.64 (3)
O^vi^—La—O^vii^	97.88 (2)	O^iv^—La—La^iv^	103.00 (3)
O^i^—La—O^viii^	68.24 (3)	O^v^—La—La^iv^	129.62 (3)
O^ii^—La—O^viii^	74.20 (2)	O^vi^—La—La^iv^	85.04 (3)
O^iii^—La—O^viii^	77.64 (5)	O^vii^—La—La^iv^	38.79 (3)
O^iv^—La—O^viii^	148.98 (6)	O^viii^—La—La^iv^	103.20 (4)
O^v^—La—O^viii^	97.88 (2)	La^ix^—La—La^iv^	83.823 (4)
O^vi^—La—O^viii^	97.88 (2)	La^x^—La—La^iv^	123.628 (3)
O^vii^—La—O^viii^	136.53 (7)	La^xi^—La—La^iv^	123.628 (3)
O^i^—La—La^ix^	66.64 (3)	O^xii^—Mo—O^xiii^	107.08 (5)
O^ii^—La—La^ix^	103.00 (3)	O^xii^—Mo—O^xiv^	114.36 (10)
O^iii^—La—La^ix^	38.85 (3)	O^xiii^—Mo—O^xiv^	107.08 (5)
O^iv^—La—La^ix^	161.84 (3)	O^xii^—Mo—O	107.08 (5)
O^v^—La—La^ix^	85.04 (3)	O^xiii^—Mo—O	114.36 (10)
O^vi^—La—La^ix^	129.62 (3)	O^xiv^—Mo—O	107.08 (5)
O^vii^—La—La^ix^	103.20 (4)	Mo—O—La^iv^	134.75 (7)
O^viii^—La—La^ix^	38.79 (3)	Mo—O—La^xv^	120.66 (7)
O^i^—La—La^x^	103.00 (3)	La^iv^—O—La^xv^	102.36 (5)
O^ii^—La—La^x^	38.85 (3)		

Symmetry codes: (i) *y*+1/4, −*x*+1/4, *z*+1/4; (ii) *x*, *y*+1/2, −*z*+1; (iii) −*y*−1/4, *x*+1/4, *z*+1/4; (iv) −*x*, −*y*, −*z*+1; (v) *x*−1/2, *y*+1/2, *z*+1/2; (vi) −*x*+1/2, −*y*, *z*+1/2; (vii) −*y*−1/4, *x*−1/4, −*z*+3/4; (viii) *y*+1/4, −*x*+3/4, −*z*+3/4; (ix) −*x*, −*y*+1, −*z*+1; (x) −*x*+1/2, −*y*+1/2, −*z*+3/2; (xi) −*x*−1/2, −*y*+1/2, −*z*+3/2; (xii) *y*−1/4, −*x*+1/4, −*z*+1/4; (xiii) −*x*, −*y*+1/2, *z*; (xiv) −*y*+1/4, *x*+1/4, −*z*+1/4; (xv) *x*+1/2, *y*−1/2, *z*−1/2.
